# Analysis of the factors influencing the dissemination of Mazu culture in Southeast Asia during the Qing Dynasty

**DOI:** 10.1371/journal.pone.0325164

**Published:** 2025-06-05

**Authors:** Dingying Lin, Zhiming Zhou, Xiaobin Zhang, Zhanhong Wu

**Affiliations:** 1 School of basic medical science, Putian University, Putian, Fujian, China; 2 Faculty of Chinese Medicine, Macau University of Science and Technology, Macao, China; 3 School of Electromechanical and Information Engineering, Putian University, Putian, Fujian, China; University of California Santa Cruz, UNITED STATES OF AMERICA

## Abstract

Mazu culture has had a profound impact on Southeast Asia. Its widespread dissemination during the Qing Dynasty resulted from the combined influence of various factors. This study uses the number of Mazu temples in Southeast Asia during the Qing Dynasty as a quantitative indicator and employs Pearson, Spearman, and Kendall correlation analyses to reveal the central role of geographical environment in cultural diffusion. The data show that there is a threshold effect between the scale of immigration and the distribution of Mazu temples: when the immigrant population exceeds 100,000, the impact significantly weakens. The propagation of Mazu culture is significantly underpinned by a distinctive geo-economic environment, notably characterized by an extensive coastline and a high coastline-to-land ratio, which inherently fosters the development of a marine economy. The high concentration of Mazu temples in port cities highlights the role of the compatibility between the maritime economic network and geographical space in promoting the dissemination of the belief. Consequently, the dissemination and spatial distribution of Mazu culture during the Qing Dynasty were intrinsically linked to the distinctive geographical environment of Southeast Asia.

## Introduction

The belief in Mazu originated during China’s Song Dynasty. Mazu was born in a fishing village on Meizhou Island in Putian. Throughout her life, she was renowned for her filial piety, assistance to those in distress, serving as a “local shaman” (a village witch who divined fortunes, acting as a medium between humans and deities in ancient beliefs), practicing medicine to save lives, and rescuing those in maritime disasters. Her benevolent actions earned her the deep affection of the people. After her passing, the local populace erected temples in her honor on Meizhou Island in Putian, Fujian, China, marking the beginning of Mazu’s establishment as a maritime protector deity [1–3].

Mazu culture centers on the worship of Mazu, with Mazu temples, rituals, myths and legends, literature, and the arts serving as its primary carriers. This distinctive culture has evolved by deriving and integrating various cultural elements. With a history that spans over a thousand years, Mazu culture has remained vibrant and enduring. Since her birth, Mazu has significantly influenced and played a vital role in political, economic, religious, cultural, social, maritime, and diplomatic spheres. Emperors throughout Chinese history have bestowed honors upon her 36 times, official titles for Mazu have amounted to 16 across different dynasties, and folk titles exceed one hundred, making her a treasured cultural gem of the Chinese nation. Today, there are over 5,000 Mazu temples worldwide, with devotees spread across 33 countries and regions, numbering more than 200 million [4,5]. On September 30, 2009, “Mazu Beliefs and Customs” was inscribed on UNESCO’s Representative List of the Intangible Cultural Heritage of Humanity, becoming China’s first intangible cultural heritage of faith and customs, signifying that Mazu culture has become a shared spiritual wealth for all humanity. Recognized globally as the goddess of maritime peace, Mazu embodies rich cultural connotations and has facilitated cross-cultural exchanges, particularly evident in her integration into East Asian regional practices. For instance, the acceptance of Mazu worship by Japanese maritime deities illustrates the fusion of local traditions with Mazu veneration, resulting in a unique cultural amalgamation [6,7]. Mazu’s influence has also extended to Vietnam and other Southeast Asian countries, where Chinese immigrants have established temples and integrated Mazu worship into local customs, promoting multicultural exchanges [8,9]. Nowadays, Mazu culture, which originated in Putian, Fujian, China, has evolved into a cultural symbol with transnational and cross-regional influence. It establishes a solid cultural foundation for China’s construction of the 21st Century Maritime Silk Road and plays a significant role in promoting cultural exchanges and fostering mutual understanding and inclusiveness among nations.

Mazu culture serves as a bridge for cross-cultural interaction and communication. As Chinese immigrants established Mazu temples in various geographical locations, which created spaces for cultural exchange and mutual understanding with local residents. This cross-cultural dialogue fostered appreciation and respect for Chinese traditions, promoted multiculturalism, and enriched global cultural diversity. Therefore, studying the dissemination of Mazu culture is crucial. By examining how Mazu worship has expanded from its origin in Putian to other parts of Asia, we can uncover the mechanisms by which cultural customs are preserved, transformed, and integrated within diverse social environments. Such research provides valuable insights into the dynamics of cultural transmission and adaptation in a globalized world. These insights are essential for understanding the processes of cultural globalization and the resilience of traditional customs in the face of rapid social change.

### Current state of Mazu culture research

Recent scholarship on Mazu beliefs has grown significantly, yielding numerous valuable findings. Existing research has broadly explored aspects such as the general patterns of Mazu belief dissemination, temple distribution, cultural characteristics, developmental trends, heritage resources, practical significance, and the impact of religious policies in the region.

Studies focusing on the macro-historical overview and trajectory have laid important groundwork. The monograph by Li TX [10] systematically compiled historical sources on Mazu beliefs within overseas Chinese communities, analyzing its global spread and cultural traits. Song JX [11] highlighted the centrality of countries along the Maritime Silk Road in Mazu culture’s expansion and examined the historical continuity of its cultural sphere. From a historical-cultural geography perspective, Zheng HB [12] identified spontaneous cultural dissemination, folk migration, and government influence as key drivers, validating their impact using spatial statistics. Lin GP *et al*. [13] emphasized the openness, inclusiveness, and adaptability of Mazu beliefs, coupled with early official recognition, as crucial factors enabling its global spread through trade and migration. Huang J’s [14] comprehensive documentation of Mazu temples provides an invaluable resource, analyzed through interdisciplinary lenses. Lin Z [15] compiled Ming and Qing dynasty accounts of Mazu’s miracles, revealing state attention and its diplomatic significance. Collectively, these works provide essential historical context, data, and analytical frameworks for understanding the broader patterns of Mazu belief propagation.

Research on cultural adaptation and localization has shed light on how Mazu beliefs integrated and evolved within specific Southeast Asian contexts. Xu YZ [16] offered an overview of Mazu worship in Southeast Asia, noting its syncretic nature (blending Buddhist and Taoist elements) and positive societal impacts. Phan THI [17] provided an in-depth look at the localization process in a specific Vietnamese village, illustrating the interplay between Chinese immigrant and local cultures. Dy AC [18] analyzed the syncretism of the Virgin Mary with Mazu and Guanyin among Filipino Chinese, attributing it to pragmatism, perceived religious universality, and a conciliatory cultural mindset. Triatmodjo S *et al*. [19], through an analysis of temple space in Indonesia, elucidated Mazu’s mythical practices and function as a tutelary deity within local Chinese and Javanese communities. Andaya BW [20] argued, using historical and anthropological evidence, that belief in deities like Mazu provided crucial intangible support for maritime peoples coping with navigational uncertainties, thus sustaining large-scale trade. James LW [21], examining Mazu culture in Hong Kong and Guangdong, proposed the concept of “standardization of deities,” linking Mazu’s status to imperial promotion in late imperial China. These studies effectively demonstrate the adaptability and diverse manifestations of Mazu beliefs across different cultural landscapes. Furthermore, some scholars have focused on theoretical conceptualization and contemporary relevance. Huang RG [22] introduced the concept of “Mazu Studies,” establishing its ontological foundation. Li HF [23] explored the impact of religious policies and Mazu culture’s potential role as a cultural bridge under the Belt and Road Initiative. Sang GR [21] applied Marxist perspectives and the concept of “spirits” to examine cultural production and temple legitimacy.

However, despite the breadth and depth of existing research, certain shortcomings remain. Firstly, while many studies describe the phenomena and characteristics of Mazu belief dissemination (such as drivers, cultural adaptation, temple distribution), a systematic and in-depth investigation specifically targeting the primary influencing factors driving its spread to Southeast Asia during the Qing Dynasty—a pivotal period for its overseas expansion—and the interplay between these factors, is notably lacking. Existing literature tends to focus either on broad historical narratives, specific case studies of individual countries or communities, or contemporary implications, without adequately concentrating on the core dynamics within the specific spatio-temporal context of Qing-era Southeast Asia. Secondly, although factors like migration, trade, and government influence are frequently mentioned, their specific manifestations, relative importance, and interaction with the local geographical environment and social structures in shaping the precise pathways and patterns of Mazu belief propagation in Qing Dynasty Southeast Asia require further nuanced investigation.

Building upon previous scholarship, this paper adopts Southeast Asia’s geographical environment as a starting point and draws upon foundational historical materials concerning Mazu culture. It focuses specifically on the crucial Qing Dynasty period to conduct an in-depth analysis of the main factors influencing the spread of Mazu beliefs to Southeast Asia and their mechanisms of action, aiming to address the identified gap in the current literature.

## Materials and methods

Mazu temples, as the primary venues for Mazu belief, comprehensively exhibit and disseminate Mazu culture through their unique architectural artistry, vibrant worship activities, rich cultural heritage, extensive historical records, and strong community connections. These temples serve not only as spaces where devotees seek protection and express reverence but also as vital institutions for preserving and promoting the spirit of Mazu, fostering community cohesion, and facilitating cultural exchange [24]. As symbols of folk beliefs, Mazu temples solidify the influence of deities across territories from both spatial and temporal perspectives. This influence is largely determined by factors such as the characteristics of worshipers, migration patterns, and the reach of their influence.

The Qing Dynasty marked a crucial period for the spread of Mazu culture across Southeast Asia. During this time, foreign trade flourished, and migration from coastal areas, particularly Fujian and Guangdong, increased. Consequently, Mazu culture gradually permeated various Southeast Asian countries through these coastal immigrants. This paper quantifies the extent of Mazu culture dissemination through the number of Mazu temples constructed in Southeast Asian countries during the Qing Dynasty. Additionally, it employs three correlation coefficient methods to calculate the correlation coefficients between the number of Mazu temples and factors such as coastline length, land area, and immigration figures to assess the primary influence factors of Mazu culture’s dissemination.

The data collected in this study on the number of Mazu temples constructed and immigrant populations in Southeast Asian countries during the Qing Dynasty is presented in Table 1. The coastline length, land area, and coastline-to-land area ratio are included to represent each country’s maritime resources and geographical complexity. The number of Mazu temples in each Southeast Asian country during the Qing Dynasty is drawn from reference [14], while immigration data is sourced from reference [25].

**Table 1:** Data related to Mazu culture in Southeast Asian countries.

Country	Land area ($10^{4}\;{\text{km}}^{2}$)	Coastline ($10^{4}$ km)	Coastline/Land area (%)	Temples	Immigrants ($10^{4}$)
Philippines	29.98	1.85	6.18	100	10.0
Malaysia	33.08	0.42	1.25	61	17.4
Indonesia	190.46	5.47	2.87	53	12.2
Vietnam	33.17	0.33	0.98	22	23.2
Thailand	51.31	0.32	0.63	20	100.0
Singapore	0.07	0.02	26.51	11	16.4
Myanmar	67.66	0.22	0.33	8	12.2
Cambodia	18.10	0.04	0.24	0	30.0

Three algorithms for calculating correlation coefficients were used to compute the correlation between the number of Mazu temples and coastline length, land area, number of immigrants, and the ratio of coastline to land area. The three correlation coefficient algorithms are :

1. Pearson correlation coefficient

The Pearson correlation coefficient (usually denoted as r) measures the linear correlation between two continuous variables. Its value ranges from –1 to 1, where:1 indicates perfect positive correlation; –1 indicates perfect negative correlation; 0 indicates no linear correlation.

The formula for the Pearson correlation coefficient is:

$$r=\frac{\sum_{i=1}^{n}(x_{i}-\bar{x})(y_{i}-\bar{y})}{\sqrt{\sum_{i=1}^{n}(x_{i}-\bar{x}{)}^{2}}\sqrt{\sum_{i=1}^{n}(y_{i}-\bar{y}{)}^{2}}}$$ (1)

Where: *x*_*i*_ and *y*_*i*_ are the ith observations of the two variables; $\bar{x}$ and $\bar{y}$ are the means of x and y respectively; n is the number of observations.

2. Spearman’s rank correlation coefficient

Spearman’s rank correlation coefficient (usually denoted as ρ) is a non-parametric method used to measure the monotonic relationship between two variables. It does not require the data to have a linear relationship or be normally distributed. The Spearman method first converts the data to ranks, then calculates the correlation between the ranks. The value of the Spearman’s correlation ranges from –1 to 1.

The formula for Spearman’s correlation coefficient is:

$$\rho =1-\frac{6\sum d_{i}^{2}}{n(n^{2}-1)}$$ (2)

Where: *d*_*i*_ is the difference between the ranks of the two variables for the ith observation; n is the number of observations.

If there are no tied ranks in the data, the following formula can also be used:

$$\rho =\frac{\sum_{i=1}^{n}(x_{i}-\bar{x})(y_{i}-\bar{y})}{\sqrt{\sum_{i=1}^{n}(x_{i}-\bar{x}{)}^{2}}\sqrt{\sum_{i=1}^{n}(y_{i}-\bar{y}{)}^{2}}}$$ (3)

Here, *x*_*i*_ and *y*_*i*_ are rank values, not the original data values.

3. Kendall’s rank correlation coefficient

Kendall’s rank correlation coefficient (usually denoted as τ) is also a non-parametric method used to measure the ordinal association between two variables.

There are several variants of Kendall’s τ, with $\tau_{b}$ being the most commonly used. Its formula is:

$$\tau_{b}=\frac{n_{c}-n_{d}}{\sqrt{\left(n_{0}-n_{1})(n_{0}-n_{2}\right)}}$$ (4)

Where: *n*_*c*_ is the number of concordant pairs (pairs where the ordering of both variables is consistent); *n*_*d*_ is the number of discordant pairs (pairs where the ordering of both variables is inconsistent); *n*_0_ = *n*(*n*−1)/2 (n is the number of observations); $n_{1}=\sum_{i}t_{i}(t_{i}-1)/2$ (*t*_*i*_ is the size of the ith tied group in X); $n_{2}=\sum_{j}u_{j}(u_{j}-1)/2$ (*u*_*j*_ is the size of the jth tied group in Y).

The value of Kendall’s τ also ranges from –1 to 1, and is interpreted similarly to Pearson and Spearman coefficients.

## Results

The Table 1 lists the construction of Mazu temples in Southeast Asian countries during the Qing Dynasty. The data indicate that the Philippines constructed the most Mazu temples during this period, while Cambodia built none. Spatially, there is no clear correlation between the construction of Mazu temples and a country’s geographic proximity to China. For instance, countries closer to China, such as Vietnam and Myanmar, built 22 and 8 temples, respectively—fewer than Malaysia (61 temples) and Indonesia (53 temples), which are located farther away. Additionally, Cambodia, despite being at an intermediate distance from China, did not construct any Mazu temples, whereas its neighbor Thailand built 20. Singapore, though the smallest country in terms of land area, constructed 11 Mazu temples during this period.

**Fig 1:**
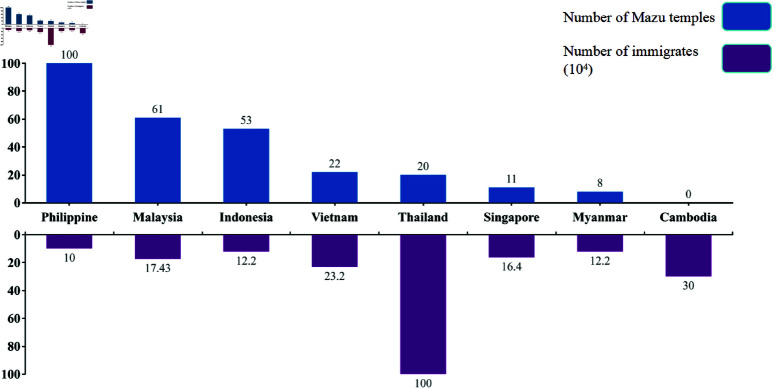
Comparison chart of Mazu temples and immigrant numbers in Southeast Asian countries during the Qing Dynasty. The blue bars represent the number of Mazu temples built in each country during the Qing Dynasty, while the brown bars represent the number of immigrants who migrated from China to Southeast Asian countries during the same period.

Traditionally, the spread of Mazu culture is considered directly linked to migration numbers. Fig 1 compares the number of Chinese migrants to Southeast Asian countries during the Qing Dynasty with the number of Mazu temples constructed in each country. The figure reveals that the Philippines, with 100,000 migrants during this period, had the highest number of Mazu temples (100), while Cambodia, despite receiving 300,000 migrants, built none, indicating a lack of proportionality between migration and temple construction. Furthermore, Thailand, which had the largest migrant population during the Qing Dynasty (1 million), had a similar number of Mazu temples as Vietnam, which had only 232,000 migrants. Notably, the three countries with the most Mazu temples—the Philippines, Malaysia, and Indonesia—each had fewer than 200,000 migrants. Conversely, the three countries with the largest migrant populations—Thailand, Cambodia, and Vietnam—ranked fifth, fourth, and last, respectively, in terms of Mazu temple construction. These observations suggest that once the migrant population exceeds 100,000, the spread of Mazu culture (as represented by the number of Mazu temples) appears less influenced by the size of the migrant population.

### Result of three correlation algorithms

**Fig 2:**
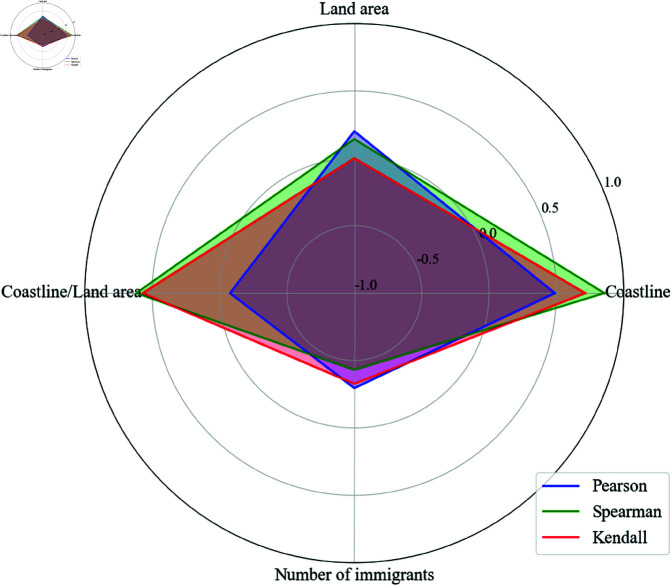
Radar chart of the results of three correlation algorithms. This chart shows the correlation coefficients between the number of Mazu temples and four variables—land area, coastline, the ratio of coastline to land area, and the number of immigrants. The blue line represents the results of the Pearson Correlation Coefficient algorithm, the green line represents the results of the Spearman’s Rank Correlation Coefficient algorithm, and the red line represents the results of the Kendall’s Rank Correlation Coefficient algorithm.

Fig 2 illustrates the results of the three correlation algorithms, with the specific calculation values and p-value validations listed in Table 2. First, the relationship between land area and the number of Mazu temples was examined. Pearson correlation analysis yielded a coefficient of 0.201 with a p-value of 0.633, indicating a weak positive correlation that is not statistically significant. Similarly, Spearman’s and Kendall’s correlation coefficients were 0.143 and 0.000, with p-values of 0.736 and 1.000, respectively, further suggesting the absence of an ordinal monotonic relationship between land area and the target variable. These findings indicate that land area has minimal influence on the number of Mazu temples. Neither linear nor non-linear ordinal relationships exhibit significant correlations, suggesting that land area is not a critical factor affecting the distribution of Mazu temples.

**Table 2:** Correlation analysis results.

Variable	Pearson (r, p-value)	Spearman (rho, p-value)	Kendall’s Tau (tau, p-value)
Land area	0.201, 0.633	0.143, 0.736	0.000, 1.000
Coastline	0.489, 0.218	0.857, 0.007	0.714, 0.014
Coastline/Land area	–0.077, 0.856	0.619, 0.102	0.571, 0.061
Number of immigrants	–0.295, 0.477	–0.431, 0.286	–0.327, 0.262

The relationship between coastline length and the number of Mazu temples shows a strong and significant correlation. Pearson correlation analysis yields a coefficient of 0.489 with a p-value of 0.218, indicating a moderate positive correlation that is not statistically significant. However, Spearman’s correlation coefficient is 0.857 (p = 0.007), and Kendall’s correlation coefficient is 0.714 (p = 0.014), both of which reveal a significant strong positive correlation. These results suggest that coastline length is a critical factor influencing the number of Mazu temples, indicating that longer coastlines are associated with more Mazu temples. This significant positive correlation is more apparent in ordinal monotonic relationships, highlighting the role of coastal geographical environments in facilitating the spread of Mazu culture and promoting the establishment of Mazu temples. Thus, coastline length is confirmed as an essential factor, consistent with the close relationship between Mazu culture and the marine environment.

The impact of the coastline-to-land area ratio is more complex. In geography, this ratio describes the topographical characteristics of a region, particularly the complexity of the coastline. A high ratio typically indicates a highly indented coastline with numerous bays, peninsulas, and islands, while a low ratio suggests a straighter coastline with simpler terrain. Pearson’s correlation coefficient is –0.077 (p = 0.856), showing almost no linear relationship. However, Spearman’s coefficient is 0.619 (p = 0.102), and Kendall’s coefficient is 0.571 (p = 0.061), indicating a moderate positive correlation trend in ordinal monotonic relationships. These findings suggest that while the coastline-to-land area ratio does not show a significant influence in linear correlations, higher ratios may imply a distribution trend of greater numbers of Mazu temples. This is because complex coastlines, while providing natural harbors, also pose navigation challenges, especially in archipelagic and fjord regions, increasing the risk of ship grounding or collision. Given the limited maritime technology during the Qing Dynasty, complex maritime environments posed significant dangers, prompting sailors and fishermen to seek psychological comfort and a sense of safety through faith in Mazu. Thus, the geographical features represented by a high coastline-to-land area ratio may have facilitated the spread of Mazu culture.

Finally, the relationship between the number of immigrants and the target variable is relatively weak. The Pearson correlation coefficient is –0.295 with a p-value of 0.477, indicating a mild negative correlation that is not statistically significant. Similarly, Spearman’s and Kendall’s correlation coefficients are –0.431 (p = 0.286) and –0.327 (p = 0.262), respectively, further suggesting a negative correlation trend that does not reach significance. Combined with the analysis of Fig 1, it is evident that once the number of immigrants reaches a certain level, an increase in immigration does not lead to an increase in the number of Mazu temples and may even have a negative impact. This phenomenon can be attributed to cultural barriers among diverse immigrant groups and conflicts between immigrant cultures and local traditional beliefs and customs, which hinder the proliferation of Mazu temples as immigrant numbers grow.

In summary, the results of the three correlation analysis methods indicate that the most significant influencing factor on the number of Mazu temples is coastline length, while the coastline-to-land area ratio is a potential influencing factor, and land area and the number of immigrants show no significant correlation. Coastline length demonstrates a significant strong positive correlation in non-linear relationships and is an important determinant of the number of Mazu temples. The coastline-to-land area ratio exhibits a positive correlation trend that, while not statistically significant, suggests a potential facilitative role. Land area and the number of immigrants have weak impacts on the target variable, with neither linear nor non-linear correlations reaching significance.

### Analysis of the impact of coastline and coastline-to-land area ratio

**Fig 3:**
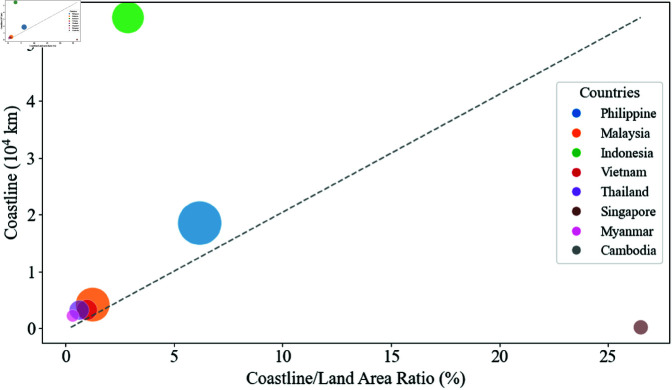
Relationship between the number of Mazu temples, coastline length, and the ratio of coastline to land area. The x-axis represents the ratio of coastline to land area, the y-axis represents coastline length, and the size of the circles indicates the number of Mazu temples, with larger circles representing a greater number of temples.

Based on the results of the three correlation algorithms, Fig 3 illustrates the relationship between the number of Mazu temples built during the Qing Dynasty in eight countries, their coastline length, and the ratio of coastline length to land area. In this figure, apart from Indonesia and Singapore, which are notable outliers deviating significantly from the dashed line, the number of Mazu temples in other countries shows an increasing trend along the dashed line. Despite Indonesia’s extremely long coastline and Singapore’s exceptionally high coastline-to-land area ratio, neither country exhibits a substantial increase in the number of Mazu temples. This indicates that the number of Mazu temples is influenced by both coastline length and the coastline-to-land area ratio, with limited impact from either factor alone.

The Philippines and Indonesia are both archipelagic nations, with the Philippines comprising over 7,000 islands and Indonesia, as the largest archipelagic country in the world, comprising over 17,000 islands. While Indonesia’s coastline is 2.95 times longer than the Philippines’, its coastline-to-land area ratio is only 46.5% of the Philippines’. From a geographical perspective, Indonesia’s extensive coastline is largely formed by five main islands and island groups. In contrast, the Philippines, with fewer islands and a shorter coastline than Indonesia, lacks large islands and is predominantly composed of small- and medium-sized islands, resulting in a higher coastline-to-land area ratio. Indonesia’s population is primarily concentrated on Java Island, with a significant portion residing in its agricultural economic zones, leaving maritime core areas relatively underpopulated. Conversely, due to the lack of large islands, the Philippines’ coastal lowlands serve as the primary population and economic zones, with its economy and culture heavily reliant on coastal resources and ports. A higher proportion of the population engaged in maritime economic activities facilitates the dissemination of Mazu culture, as seafaring activities often face natural challenges and threats. During the Qing Dynasty, maritime technology was insufficient to overcome these difficulties and natural disasters, prompting sailors to turn to Mazu worship for divine protection. Furthermore, a high coastline-to-land area ratio means more coastal communities are exposed to typhoons, tsunamis, and other natural disasters, increasing the public acceptance and significance of Mazu belief. These geographical differences explain why the Philippines, with fewer immigrants, a shorter coastline, and a smaller land area than Indonesia, has a greater number of Mazu temples, demonstrating that the spread of Mazu culture is influenced by both coastline length and the coastline-to-land area ratio. An appropriate coastline length and coastline-to-land area ratio represent specific geographical characteristics conducive to a maritime economy. These features can foster a greater number of maritime economic participants, thereby facilitating the dissemination of Mazu culture.

Although Singapore has an extremely high coastline-to-land area ratio, its actual coastline length is very short. The limited national land area leads to saturation in the number of Mazu temples within the available space, making further increases difficult, which explains the relatively low number of Mazu temples in Singapore. Other countries, such as Vietnam, Thailand, Myanmar, and Cambodia, have relatively short coastlines and low coastline-to-land area ratios, resulting in fewer or no Mazu temples. This indicates that when a country’s coastline length and coastline-to-land area ratio are insufficient to support frequent maritime activities, the demand and motivation for establishing Mazu temples are correspondingly weakened. The decline in maritime trade and fishing activities has curtailed the dissemination of maritime-related cultures and beliefs.

Examining the number of Mazu temples in Malaysia and Indonesia reveals Malaysia as a unique case among Southeast Asian countries. Compared to Indonesia, Malaysia has both a shorter coastline and a lower coastline-to-land area ratio. However, Malaysia surpasses Indonesia in the number of Mazu temples. This anomaly is related to Malaysia’s strategic position as a critical node in Southeast Asian trade during the Qing Dynasty. As a trade hub, Malaysia hosted several key ports, and port trade facilitated the dissemination of Mazu culture along its coastal areas. Therefore, despite Malaysia’s less advantageous geographical characteristics, its location in Southeast Asia established it as a trade hub, strengthening its maritime economic attributes and promoting the dissemination of Mazu culture. This demonstrates that geographical features conducive to maritime economies depend not only on intrinsic geographical characteristics but also on a country’s global geographical position, which explains the statistical insignificance of the coastline-to-land area ratio.

To further verify the impact of the marine economy on the dissemination of Mazu culture, based on the reference [14], Tables 3, 4, 5, 6, 7, 8, 9 present the distribution of Mazu temples in Southeast Asia during the Qing Dynasty. Firstly, based on the data in the tables, it is evident that Mazu temples are predominantly distributed along the coast, also extending to inland water systems. The distribution of these temples exhibits a clear coastal characteristic, as the vast majority of the temples are located in coastal cities or islands. This is closely related to Mazu’s role as the Sea Goddess. Secondly, the spatial distribution of Mazu temples in Southeast Asia is highly correlated with port cities and trade centers. Nearly all of the Mazu temples are situated in historically or currently important ports and trade hubs, whether they are major international ports, regional ports, or even inland river ports. Only a few Mazu temples are located in inland cities. Cities with a concentration of Mazu temples, such as Batangas City in the Philippines; Kuala Lumpur, Klang, George Town, Malacca City, and Sandakan in Malaysia; Medan, Tanjung Pinang, Jambi, and Semarang in Indonesia; Ho Chi Minh City and Hoi An in Vietnam; and Bangkok in Thailand, have historically been significant ports and trade cities. Combined with the immigration data from Fig 1, it suggests that the spatial distribution of Mazu temples in Southeast Asian countries during the Qing Dynasty was the result of a combination of migration, trade, and geographical factors. Immigration was the direct driving force, with Chinese immigrants bringing the Mazu belief to Southeast Asia and establishing temples in their settlements. Trade played an important role in accelerating the spread of the Mazu belief, with port cities becoming concentrated areas for Mazu temples. The geographical environment provided the basic conditions, as the coastal geography and river systems of Southeast Asia offered spatial carriers for the distribution of Mazu temples.

**Table 3:** Mazu temples in Malaysia.

State/Province/Prefecture	City/County	Mazu Temple Count	City characteristic
Selangor	Kuala Lumpur City	4	Port City Cluster
	Pulau Ketam	1	Island (Natural Port)
	Klang City	1	Port City
Pahang	Kuantan City	2	Port City
	Lipis	1	Inland City
	Temerloh	1	Inland City
Sarawak	Kuching City	6	Port City
Sabah	Sandakan	1	Port City
Penang	George Town	8	Port City
	Butterworth	2	Port City Cluster
	Balik Pulau	2	Inland City
Malacca	Malacca City	6	Port City
Johor	Batu Pahat	3	Port City
	Muar	3	Port City
	Kulai	1	Inland City
	Kota Tinggi	1	River City
	Johor Bahru	1	Port City
Kelantan	Kota Bharu	3	Near Coastal City
	Pasir Puteh	1	Inland City
Terengganu	Kuala Terengganu	3	Port City
	Kuala Nerus	1	Near Coastal City
	Kemaman	1	Near Coastal City
Perak	Taiping	2	Port City
	Teluk Intan	1	River Port City
Others		5	
Total		61	

**Table 4:** Mazu temples in Indonesia.

State/Province/Prefecture	City/County	Mazu Temple Count	City characteristic
North Sumatra	Medan City	2	Near Belawan Port
	Tanjung Balai City	1	Port City
Riau	Tanjung Pinang City	3	Port City
	Binjai City	1	Inland City
	Jakarta City	2	Port City
Yogyakarta	Yogyakarta City	1	Inland City
West Java	Cirebon City	1	Port City
	Semarang City	3	Port City
	Demak City	2	Near Coastal City
	Pekalongan City	1	Near Coastal City
	Solo City	1	Inland City
	Rembang City	4	Near Coastal City
	Surabaya City	2	Port City
East Java	Madiun City	2	Inland City
	Gresik City	2	Port City Cluster
West Kalimantan	Pontianak City	2	River Port City
	Mempawah City	2	Port City
	Sambas City	2	Port City
	Singkawang City	2	Port City
	Samarinda City	1	River Port City
South Sulawesi	Makassar City	6	Port City
	Madura Island	2	Island (Natural Port)
Others		4	
Total		53	

**Table 5:** Mazu temples in Vietnam.

State/Province	City/County	Mazu Temple Count	City characteristic
Dong Thap	Sa Dec	1	River Port City
Ho Chi Minh City	Ho Chi Minh City	9	Port City
Hanoi	Hanoi	1	River City
Quang Nam	Hoi An	10	Port City
Dong Nai	Bien Hoa	1	Port City Cluster
Total		22	

**Table 6:** Mazu temples in Singapore.

City/County	Mazu Temple Count	City characteristic
Singapore	11	Port City
Total	11	

Therefore, after the number of immigrants exceeds a certain threshold, the scale of the immigrant population no longer serves as the primary driving force for the dissemination of Mazu culture. Instead, specific geographic features conducive to the development of the maritime economy become the key factors. These geographic features include a vast coastline and a higher ratio of coastline to land area. The extensive coastline provides more opportunities for maritime activities, while the higher coastline-to-land-area ratio signifies a moderate concentration of coastal populations and the prosperity of maritime economic activities. This, in turn, facilitates the emergence of more participants in the maritime economy, thereby further promoting the spread of Mazu culture.

**Table 7:** Mazu temples in Myanmar.

State/Province	City/County	Mazu Temple Count	City characteristic
Tanintharyi Region	Myeik	2	Port City
Yangon Region	Yangon	2	Port City
Ayeyarwady Region	Pathein	1	Port City
Bago Region	Taungoo	2	Inland City
Total		7	

**Table 8:** Mazu temples in Thailand.

State/Province	City/County	Mazu Temple Count	City characteristic
Bangkok	Bangkok	5	Port City
Surat Thani	Surat Thani	3	Port City
Nakhon Si Thammarat	Nakhon Si Thammarat	3	Port City
Phuket	Phuket	2	Island (Natural Port)
Ranong	Ranong	1	Port City
Prachinburi	Prachinburi	1	Inland City
Prachuap Khiri Khan	Prachuap Khiri Khan	1	Port City
Sing Buri	Sing Buri	1	Inland City
Lopburi	Lopburi	1	Inland City
Nakhon Pathom	Nakhon Pathom City	2	Inland City
Songkhla	Songkhla	1	Port City
Total		20	

**Table 9:** Mazu temples in Philippines.

City/County	Mazu Temple Count	City characteristic
Batangas City, Lipa City, Tanauan City, Rosario Municipality *et al*.	100	Port City/Port City Cluster
Total	100	

## Discussion

The spread of Mazu culture is deeply rooted in specific geographical environments, closely connected to its nature as a maritime culture. Maritime culture encompasses the material and spiritual civilization created by humans through long-term exploration, understanding, and utilization of the ocean. Since ancient times, the ocean has symbolized both mystery and challenge, while also offering resources and opportunities for humanity. The prosperity of the Maritime Silk Road during the Song Dynasty not only facilitated economic and cultural exchanges between East and West but also provided a material and technological foundation for cross-sea trade and the development of navigation techniques. Over time, influenced by local customs and natural environments, people gradually formed distinct regional folk beliefs related to the sea. These beliefs reflect humanity’s imagination and response to the ocean’s power, especially when facing its unpredictable and perilous nature. Worshipping deities became a crucial means for seeking psychological comfort and security. Given the limitations of navigation technology and oceanic knowledge at the time, people urgently needed spiritual support to face the risks and challenges of the sea.

Mazu was born in Putian City, Fujian Province, located along the southeastern coast of China. This region boasts a superior geographical position, nestled against the majestic Daiyun Mountain Range and facing the vast Pacific Ocean. It features a long and intricate coastline, complemented by numerous deep-water natural harbors. This unique geographical environment has fostered a highly developed shipbuilding and maritime industry, making it a pivotal base for ancient maritime trade. Meizhou Island, an important island along Putian’s coastline, has been home to generations of residents who earn their living through fishing and seafaring. Living in the tumultuous and frequently stormy marine environment, these inhabitants often face threats and challenges posed by natural conditions. Confronted with fears and uncertainties about the unknown, as well as concerns for their survival and safety, they naturally sought the protection and blessings of supernatural forces and deities. Consequently, they carried Mazu’s statues, talismans, and other symbolic items to pray for safety during their voyages. The “Records of the Investiture of Empress Tianhou” document the strong need for sea deities among the populace during maritime crises: “In times of extreme peril, they would pray to the Heavenly Consort, as if descending from the sky, with divine light shining brightly and a unique fragrance wafting, transforming the raging sea storm into a gentle breeze” [26]. Through this intense yearning for an idealized deity and the practice of faith, Mazu gradually established her esteemed status as a maritime protector deity, continually gaining recognition and strengthening the faith of coastal communities.

As an important component of marine civilization, the historical records and related myths and legends of Mazu culture are closely associated with maritime activities. These rich accounts greatly enrich the connotations and extensions of Mazu culture. Mazu is widely regarded as the guardian deity of seafaring. Before each voyage, rituals are performed to honor Mazu, praying for safe and smooth journeys. The Song Dynasty poet Liu Kezhuang’s “Twenty Rhymes of Baihu Temple” meticulously documents the spread and development of Mazu belief during the Song Dynasty, reflecting the widespread influence of Mazu worship in society at that time [27]. Similarly, the Ming Dynasty’s “Record of Missions to Ryukyu” recounts how Ryukyu envoys, during their return voyages, sought Mazu’s protection when encountering severe risks at sea, illustrating the dissemination and impact of Mazu belief in overseas regions [28]. In the Qing Dynasty, the poet Cui Xu described in his works the miraculous experiences of Mazu dressed in vermilion garments, safeguarding maritime navigation, and being honored with the title “Heavenly Consort,” receiving widespread worship and veneration from the populace [29]. Additionally, Qing Dynasty poet Xie Xixun, in his poem “Fujian Boat Voyage,” vividly portrays scenes where sailors fervently pray to Mazu for safety when facing storms and swollen streams [30]. These literary works and historical records not only reflect the spread and influence of Mazu belief across different historical periods but also demonstrate the close connection between Mazu culture and maritime activities.

The birthplace of Mazu culture and its related historical records indicate that Mazu culture has been closely linked with maritime activities since its inception. The origin region of Mazu culture boasts an extensive coastline, a developed shipbuilding industry, and a thriving maritime trade, reflecting the high population density in coastal areas and a deep reliance on marine resources. This aligns closely with the present study’s proposition that the length of the coastline and its ratio to land area are significant geographical factors influencing the dissemination of Mazu culture. A long and intricate coastline not only provides natural conditions favorable for seafaring activities but also creates advantageous pathways and platforms for the spread of Mazu belief. Historical documents recounting Mazu’s deeds are all related to maritime activities, further illustrating that Mazu culture has been continuously reinforced and propagated through prolonged maritime practices. Therefore, the marine environment and geographical conditions have played a crucial role in the formation and dissemination of Mazu culture, serving as important dimensions for understanding the cultural connotations and influence of Mazu.

In addition to the influence of marine environments and geographical conditions, this paper further explores the impact of technological development, warfare, and the local residents of Southeast Asia’s acceptance of Mazu culture on the spread of Mazu culture. Firstly, since its inception, Mazu has been closely associated with fishermen and other seafarers, which means her sphere of influence easily expanded to all places accessible by boat from her hometown in Fujian Province. In other words, the networks of fishermen, merchants, and others also served as networks for religious practices, bringing Mazu to new locations [31]. Thus, the spread of Mazu culture in Southeast Asia during the Qing dynasty was closely related to the maritime navigation technologies of the time. For example, trade between China and Batavia (modern-day Jakarta) in the 17th and 18th centuries saw Chinese sailing ships carrying goods aimed at the Indonesian market, which contributed to the prosperity of Batavia [32]. The peak of sailing trade occurred between 1690 and 1730, yet most observers expressed concerns about the seaworthiness of Chinese sailing vessels. Stavorinus JS argued that the design of the sailing ships was ill-suited to withstand stormy seas [33]. Ong T, in comparing them to European ships, contemptuously remarked, “From Xiamen, our sailing ships look crude, fixed with straw, and they resemble mere toys for children” [34]. Due to the relative backwardness of maritime technology and the inability to cope with harsh sea conditions, crew members would set up altars on board to worship Mazu, seeking safety during voyages. In the late 19th century, with technological advancements and social development, mechanical propulsion and advanced navigational equipment replaced sailing ships in maritime transportation, significantly enhancing navigation safety and reducing maritime accidents. Mazu’s divine protection over maritime safety weakened, but people still fervently believed that she would bless them with prosperity and wealth, thus Mazu came to be viewed not only as the protector of maritime affairs but also as a protector of business [35]. Therefore, although the development of maritime technology diminished Mazu’s role as a sea goddess, it fostered the development of her associations with business protection, wealth, and benevolence.

Secondly, immigration is the direct driving force behind the spread of Mazu culture. Immigration is not a singular societal phenomenon; its causes are complex and multifaceted, closely linked to trade-driven economic activities and deeply influenced by political turbulence and military conflict. During the late Qing period, major historical events such as the Taiping Rebellion and the Opium Wars led to significant social and economic upheaval, which directly facilitated the large-scale migration of residents from the southeastern coastal regions of China to Southeast Asia, known as the “migration to the South Seas.” In Li TX’s The Mazu Belief of Overseas Chinese from Chaoshan and Thailand, the focus is on two waves of migration from Fujian and Guangdong to Thailand, one during the late Qing and the other after World War II [36]. These migrant groups were not only spatially displaced but were also carriers of culture and belief. They brought the important cultural symbol of their homeland—Mazu belief—into Southeast Asia, where it was passed down and reproduced in the new social context, thereby establishing new cultural communities and identity ties in foreign lands.

Lastly, the local acceptance of Mazu culture in Southeast Asia also significantly affected its spread, as demonstrated by a comparison between Penang and Phuket [37]. Penang, as a multicultural port city, had a more diverse social structure, with Islam, Christianity, Hinduism, and other religions coexisting. In this environment, Mazu culture had a relatively weaker influence in public life, being preserved more as a cultural heritage. In contrast, Phuket, as an island city, had Mazu worship closely linked to the local fishing community and maritime trade. Mazu not only represented the sea god but also symbolized the guardian of the entire island, giving Mazu belief a broader and deeper influence in the local society.

In summary, the spread of Mazu culture is not a simple, linear process driven by a single factor; rather, it is a dynamic system shaped by the complex interplay of multiple factors, including geographic environment, the development of maritime technology, immigration, and the local acceptance of Mazu culture. The geographic environment, as the fundamental foundation for the nurturing and development of Mazu culture, not only shaped its inherent maritime cultural traits but also laid the structural groundwork for its spatial distribution and primary transmission paths. The long, winding coastline and developed maritime economy provided the initial ecological and cultural soil for the rise of Mazu faith, as well as the geographical channels and socio-economic networks for its subsequent cross-regional transmission. The evolution of maritime technology, while somewhat adjusting the functional focus of Mazu faith—from a purely maritime rescue role to a more diversified commercial protection—remains profoundly influenced by geographic space and maritime routes in terms of its application scenarios and scope of impact. Mass population migrations driven by political, military, and other factors, especially the historical wave of migration southward, undoubtedly served as the direct impetus for the cross-cultural dissemination of Mazu culture. However, the direction of migration, the choice of settlement locations, and the establishment and development of Mazu faith in new migration destinations are all embedded within specific geographic contexts. Lastly, the varying levels of acceptance of Mazu culture across different socio-cultural environments in Southeast Asia are not isolated phenomena, but are interrelated with factors such as regional geographic location, the level of development of maritime economies, and local cultural traditions. Together, these factors have shaped the localized forms of Mazu culture’s transmission.

Therefore, a thorough investigation of the dissemination of Mazu culture must focus on the intrinsic role of the geographic environment, treating it as a core dimension for understanding the logic of Mazu culture’s emergence, transmission mechanisms, and regional differences. Only in this way can we more comprehensively and profoundly reveal the spatial diffusion patterns and cultural adaptation strategies of Mazu culture in the process of globalization, offering a theoretically valuable case for understanding the geographical effects on cultural transmission.

## Conclusion

The spread of Mazu culture in Southeast Asia is essentially a historical process shaped by the interaction between geographical environmental factors and human elements. Research shows that the specific geographical conditions, characterized by a vast coastline and a high ratio of coastline to land area, which facilitate the development of maritime economies, played a key role in the spread of Mazu culture. Immigration, though an initial driving force for cultural dissemination, exhibits a threshold effect—when the immigrant population exceeds 100,000, geographical adaptability replaces population size as the dominant factor. The multidimensionality of the geographical environment is further reflected in the hub status of port cities (such as the trade node function of Malaysia), and territorial spatial features (such as Indonesia’s long coastline but vast inland areas), which directly constrain the distribution density of Mazu temples. Meanwhile, advancements in maritime technology have promoted a functional transformation of Mazu worship, while the cultural ecology of local Southeast Asian societies (such as the coexistence of multiple religions or reliance on fisheries) determines the depth of belief penetration. This complex coupling of geographical and cultural factors not only explains the spatial heterogeneity of Mazu culture’s spread but also provides a theoretical framework for understanding the diffusion mechanisms of maritime folk beliefs. Geographical environment, by limiting economic networks, technological applications, and cultural adaptation strategies, remains an essential and foundational dimension that cannot be ignored in the study of cultural dissemination.
